# Social capital and health in a national cohort of 82,482 Open University adults in Thailand

**DOI:** 10.1177/1359105310386264

**Published:** 2011-05

**Authors:** Vasoontara Yiengprugsawan, Suwanee Khamman, Sam-ang Seubsman, Lynette L-Y Lim, Adrian C. Sleigh, the Thai Cohort Study Team

**Affiliations:** 1 National Centre for Epidemiology and Population Health, The Australian National University, Canberra, Australia; 2 National Economic and Social Development Board, Bangkok, Thailand; 3 School of Human Ecology, Sukhothai Thammathirat Open University, Nonthaburi, Thailand

**Keywords:** social capital, social trust, self-assessed health, psychological health, Thailand

## Abstract

We report associations between social capital and health among 82,482 adults in a national cohort of Open University students residing throughout Thailand. After adjusting for covariates, poor self-assessed health was positively associated with low social trust (OR = 1.88; 95% CI 1.76–2.01) and low social support (OR = 1.79; 95% CI 1.63–1.95). In addition, poor psychological health was also associated with low social trust (OR = 2.52; 95% CI 2.41–2.64) and low social support (OR = 1.80; 95% CI 1.69–1.92). Females, elderly, unpartnered, low income, and urban residents were associated with poor health. Findings suggest ways to improve social capital and heath in Thailand and other middle-income countries.

## Introduction

For more than a decade, social capital has been intensely studied in diverse settings as a determinant of human welfare, including health and education. Social capital has received considerable attention in science and policy because research results suggest that it may have a positive impact on the well-being of individuals and nations. Social capital includes not only social networks and social participation but also social trust and reciprocity (Abbott and Freeth, 2008). Informal education, lower crime, and civic engagement are all stimulated by social capital (Kawachi et al., 1999). Other studies on social capital and health have also addressed income inequality in multiple countries (Mansyur et al., 2008) and ethnic discrimination in Sweden (Lindstrom, 2008) and reported complex relationships that varied according to cultural and historical contexts. Some studies have demonstrated the variable impact of social capital on physical and psychological health in rural and urban areas (Yip et al., 2007; Ziersch et al., 2009), in subpopulations such as the elderly (De Souza and Grundy 2007; Nummela et al., 2008) or adolescents (Almgren et al., 2009; Morgan and Haglund, 2009). Overall the relationship of social capital and health is generally positive but the specific features vary from one setting to another.

There is now a mature literature linking social capital to a variety of health outcomes and well-being indicators (Kawachi et al., 2008). The links to psychological health are extensively documented (Almedom, 2005; Berry and Welsh, 2010; De Silva et al., 2005; McKenzie et al., 2002; Phongsavan et al., 2006). Other studies have revealed the utility of social capital for health promotion (Hawe and Shiell, 2000), positive effect of social participation in physical activity (Yun et al., 2010), and the role of social capital in improving access to health care (Mohseni and Lindstrom, 2007; Perry et al., 2008; Pitkin Derose and Varda, 2009).

The foregoing information is almost entirely based on studies in developed countries including Europe, North America, and Australia. There are very few studies on social capital and health and well-being in Asia, and of those most have focused on the richer countries of East Asia (Fujisawa et al., 2009; Tsunoda et al., 2008; Yamaoka, 2008). Thus there is a need for studies on social capital in the emerging countries of Asia, especially now that we know the importance of culture, history, and context when evaluating the links between social capital and health.

Thailand is a country in Southeast Asia which has gone through rapid economic growth, economic crisis and steady economic recovery during the last few decades. Most social capital in Thailand derives from family and community non-formal safety nets, especially obvious in rural areas (Khamman, 2008; World Bank, 2000). Little is known about social capital and its effect in such a diverse developing country setting. Accordingly, we have studied the association between social capital and health in Thailand. The study is based on a large national cohort of 87,134 distance learning Open University adult Thais residing throughout the country. We examine social trust and social interaction (i.e. cognitive and structural dimensions of social capital) and their relation to overall health and psychological health. The findings will be useful in identifying ways to improve social capital and heath among various population subgroups in Thailand and other middle-income countries.

## Methods

### Study population and data collection

Data were derived from 87,134 students from the Sukhothai Thammathirat Open University (STOU) who completed a baseline survey in 2005. Details on population selection and methodology have been reported elsewhere (Sleigh et al., 2008). The baseline questionnaires containing information on individual and household characteristics were sent out to approximately 200,000 STOU students. There was no coercion to participate in the study and the STOU President and research investigators reassured participants of confidentiality with participation having no influence on academic progress. The response rate was 44 per cent. The overall cohort represents the geo-demographic, ethnic, occupational and socioeconomic status of the adult Thai population: 45 per cent were male, the median age was 29 years, 31 per cent were married at enrolment, and 95 per cent were Buddhist. However, they are better educated than the general Thai population and thus are able to respond to complex health questionnaires.

The questionnaire covers a wide range of information from demographic, socioeconomic, and geographic information to health status, use of health services, risk behaviours, injuries, dietary intake, and family background. A periodic newsletter related to the Thai health-risk transition was sent to participants to keep them informed of interesting results emerging from the study. A four-year follow-up was conducted in 2009 (response rate over 70%) and the next one is due in 2013.

In this study, to prevent the influence of biologically determined good health and ill health expected at age extremes, we restrict the analysis to those aged between 20 to 49 years resulting in 82,482 respondents (those aged less than 20 years and over 50 years were relatively few in number and they are likely to have very different health outcomes and social capital compared to adults in their 20s to 40s). Explanatory variables include sex, age (20–29, 30–39, 40–49 years), marital status (married, not-married, separated, divorced, or widowed), income per month in Thai Baht: < 7,000; 7000–10,000 and >10,000 (40Baht = 1USD in 2005), and life course residence based on residence at age 12 years of age and at present (rural, rural to urban, and urban).

Ethics approval was obtained from Sukhothai Thammathirat Open University Research and Development Institute (protocol 0522/10) and the Australian National University Human Research Ethics Committee (protocol 2004344). Informed written consent was obtained from all participants.

### Measures of social capital

There is no generally accepted instrument for measuring social capital. In this study, we define and measure social capital according to cognitive (what people feel as social trust) and structural dimensions (what people do for social interaction). Because social capital generates social support, we also use social support as a measure of overall social capital. The 15 social capital questions asked in the baseline questionnaire are listed below and relate to social trust, social interaction and social support.

*Social trust question*: ‘Generally speaking, would you say that most people can be trusted?’ The responses were ‘most people can be trusted’ or ‘you cannot be too careful’. The latter category was used as a proxy of low social trust.
*Social interaction questions*: ‘How frequently do you have social interaction with (the following): (1) parents or other relatives; (2) neighbours; (3) other friends; (4) colleagues from work; (5) temple, mosque or other place of worship; (6) sports club, voluntary or service organization; (7) political parties, trade unions, environmental groups?’ Possible responses to each question were ‘every day’, ‘nearly or every week’, ‘1–2 times per month’, ‘very few’, and ‘never’. Those who responded ‘very few’ or ‘never’ were classified as having ‘low’ level of social interaction for that question.
*Social support questions*: ‘How would you rate the support you are getting from (the following): (1) family; (2) neighbours or local people; (3) other friends; (4) employer; (5) others in the workplace; (6) local government officials; (7) religious group?’ Possible responses for each question were: ‘very little support’, ‘a little support’, ‘quite a bit of support’, and ‘a lot of support’. Those who responded ‘very little’ or ‘little’ were classified as having ‘low’ level of social support for that question.

### Measures of health outcomes and risks

We have dichotomized all health outcomes and health behaviours in order to simplify analysis and interpretation of the results as well as to facilitate comparisons. Self-reported overall health on a 6-point scale is divided into ‘poor’ vs. ‘non poor’. Self-reported psychological health on a 5-point scale also is divided into two categories (‘all or most of the time’ vs. ‘some, little or none of the time’). Specific ‘yes–no’ chronic diseases health outcomes are intrinsically dichotomous. Health behaviours (smoking and drinking) were reported as ‘yes–no’.

Self-reported overall health is based on the first question of the Medical Outcomes short form instrument (SF8) – ‘Overall how would you rate your health during the past four weeks (excellent, very good, good, fair, poor, or very poor)’. For analysis, we combined the last two categories as ‘poor or very poor’ self-assessed health.

Psychological health was assessed using the two anxiety questions and one depression question of the standard Kessler 6 psychological distress questions. The questions we used were: ‘In the past 4 weeks, about how often did you feel: (1) nervous; (2) restless or fidgety; (3) everything was an effort’. Answers to each of these three questions each ranged on a 5-point scale from ‘all of the time’ to ‘none of the time’. Those who answered ‘all of the time’ or ‘most of the time’ on at least two of the three questions had their psychological health classified as ‘poor’ and others were classified as ‘non-poor’. The other three Kessler questions related to deep levels of depression (inconsolable sadness, hopeless or worthless) and were not used in this study because of concerns that people in such a state would not respond to a mailed questionnaire.

Other dichotomous ‘yes–no’ heath outcomes include doctor diagnosed chronic illness (diabetes, high cholesterol, hypertension, cancers, goitre, epilepsy, arthritis, asthma or chronic infection).

As well, dichotomous ‘yes–no’ health risk behaviours were assessed including current smoking or alcohol drinking.

### Statistical analysis and model selection

Individuals with missing data for given analyses were excluded thus totals vary slightly according to the information available. Because missing data usually involved only about 1 per cent of observations there was no need to impute values. Given the large size of our dataset, our results were stable and not affected by missing data.

We assessed the association between outcomes and potential determinants using logistic regression, reporting Odds Ratios (ORs) and *p*-values (two-tailed tests, Stata software). We followed the analytical approach of Nieminen et al. (2010) by progressively adding clusters of confounders so building a transparently adjusted final model that included all covariates.

*Model 1* reports bivariate association between the health outcomes (overall health or psychological health) and variables representing individual characteristics, social capital, chronic illness and health risk behaviours. *Model 2* reports the associations between health outcomes (overall health or psychological health) and the social capital variables adjusting for variables representing individual characteristics. *Model 3* reports the associations between health outcomes (overall health or psychological health) and the chronic illness and health risk behaviours adjusting for variables representing individual characteristics.

*Model 4* reports the associations between health outcomes (overall health or psychological health) and all the above explanatory covariates assessed together.

## Results

### Characteristics of the cohort members by sex

Half of male respondents were married compared to 37.4 per cent among females (Table 1). There were slightly more females than males, especially in the younger age group (20–29 years). Males were generally socioeconomically better off than females as reported by income per month. Less than one-third of respondents resided in rural areas and more than one-third had moved from rural to urban areas after the age of 12 years.

**Table 1.** Attributes of Thai cohort study members 2005

	Total	Males (%)	Females (%)
**Individual characteristics**			
Sex	82,482	44.9	55.0
Age in years			
20–29	44,207	45.5	60.2
30–39	27,309	37.1	29.9
40–49	10,948	17.4	9.9
Marital status			
Married	35,488	50.0	37.4
Not married	42,262	44.9	56.3
Separated, divorced, widowed	3,381	3.3	4.8
Income/month in Baht			
< 7,000	33,161	34.1	45.2
7,001–10,000	25,071	23.4	23.9
> 10,000	27,842	40.1	28.6
Residence age 12 years and at present			
Rural residents	35,882	45.0	42.3
Rural to urban areas	29,636	36.3	35.6
Urban residents	16,119	17.6	21.2
**Social capital measures**			
Social trust			
Be cautious with others	30,911	36.7	38.1
Social interactions			
Very few or never	14,034	18.0	16.2
Social support			
Little or very little support	7,465	9.9	8.4
**Health outcomes**			
Self-assessed health			
Very poor or poor	3,805	3.9	5.2
Psychological distress			
All or most of the time	8,787	9.9	11.3
**Other health covariates**			
Chronic illness^a^			
Yes	25,665	34.9	23.0
Health-risk behaviours			
Regular smoker	8,469	21.6	1.0
Regular alcohol drinker	3,981	9.9	0.7

*Note*: ^a^ Doctor diagnosed conditions including diabetes, high cholesterol, hypertension, cancers, goitre, epilepsy, arthritis, asthma or other chronic infections.

Social capital measures in this study were: low social trust (‘you cannot be too careful’), low social interactions (‘very few or never’) and low social support (‘little or very little support’). A slightly higher proportion of females than males reported low social trust, but the opposite was true for social interaction and social support. Health outcomes, measured by self-assessed overall health and psychological health, were found to be worse among females (3.9% compared to 5.2% for poor self-assessed overall health; and 9.9% and 11.3% for poor psychological health). We have also reported other health covariates which were more common among males, including one or more chronic illnesses (34.9% compared to 23.0%) and health-risk behaviours such as smoking (21.6% compared to 1.0%) and alcohol drinking (9.9% compared to 0.7%).

### Social capital and health outcomes by characteristics of cohort members

Those aged 40 to 49 years tended to report lower social capital and lower health outcomes compared to their younger peers (Table 2). Compared to their married counterparts, separated, divorced or widowed cohort members reported even lower social capital and worse health outcomes. Geographically, those who had moved from rural to urban areas since age 12 years were more likely to have low social capital. But those urbanized since childhood were more likely to report worse health outcomes. Those who reported chronic illnesses were both much more likely to report worse social capital and worse self-assessed and psychological health outcomes. Regular smokers and alcohol drinkers reported lower social support but more social interactions than those who did not smoke or drink; however these risk behaviours were more common among those with poorer health outcomes.

**Table 2.** Social capital and health outcomes by characteristics of Thai cohort study members

	Social capital			Health outcomes	
	Low trust	Low social interaction	Low social support	Poor self-assessed health	Poor psychological health
**Individual characteristics**					
Sex					
Males	36.7	18.0	9.9	3.9	9.9
Females	38.1	16.2	8.4	5.2	11.3
Age in years					
20–29	33.4	16.6	8.2	3.9	7.2
30–39	37.3	17.2	9.9	4.5	9.1
40–49	38.6	18.1	10.2	4.9	12.5
Marital status					
Married	37.2	15.0	8.4	4.3	9.1
Not married	37.3	18.4	8.9	4.7	11.2
Separated, divorced, widowed	43.0	19.2	17.3	6.5	12.3
Income/month in Baht					
< 7,000	38.3	14.6	9.6	4.9	12.0
7,001–10,000	38.1	20.2	8.9	4.7	10.4
> 10,000	36.1	17.8	8.4	4.2	9.1
Residence at age 12 and at present					
Rural residents	34.2	12.3	7.7	4.0	9.9
Rural to urban areas	40.3	26.9	10.5	5.0	11.1
Urban residents	39.8	9.6	9.5	5.3	11.7
***Other health covariates***					
Chronic illness^a^					
No	36.1	16.5	8.5	3.5	9.5
Yes	40.6	18.2	10.2	7.1	13.3
Health-risk behaviours^b^					
Smoking					
Not a regular smoker	36.4	18.8	9.7	3.6	9.5
Regular smoker	37.5	15.1	10.7	5.9	11.3
Alcohol drinking					
Not a regular alcohol drinker	36.5	18.9	9.7	3.7	9.6
Regular alcohol drinker	38.4	9.9	11.6	5.6	12.5

*Notes*: ^a^ Doctor diagnosed conditions including diabetes, high cholesterol, hypertension, cancers, goitre, epilepsy, arthritis, asthma or other chronic infections; ^b^ restricted to males due to very low rates among females.

### Social capital, self-assessed health and psychological health

The bivariate results (Model 1) show that being female, elderly, unpartnered, poor, and urban was associated with poor self-assessed and poor psychological health (Tables 3 and 4). Low social trust, low social interaction, and low social support was also associated with poor self-assessed and poor psychological health. For bivariate analysis of other health covariates, chronic illness and regular alcohol consumption was associated with poor self-assessed and poor psychological health. Most of the above bivariate associations were statistically significant.

**Table 3.** Odds Ratios relating to poor self-assessed health and social capital adjusting for individual characteristics, and health covariates

	Model 1 bivariate	Model 2 + social capital	Model 3 + health covariates	Model 4 adjusting for all covariates	95% Confidence Interval for Model 4
**Individual characteristics**					
Sex					
Males	1	1	1	1	
Females	1.38***	1.34***	1.48***	1.50***	(1.39–1.62)
Age in years					
20–29	1	1	1	1	
30–39	1.15***	1.10	1.28***	1.25***	(1.10–1.39)
40–49	1.25***	1.16*	1.44***	1.40***	(1.23–1.58)
Marital status					
Married	1	1	1	1	
Not married	1.10**	1.01	1.02	1.04	(0.96–1.11)
Separated, divorced, widowed	1.55***	1.35***	1.45***	1.31***	(1.13–1.53)
Income/month in Baht					
< 7,000	1.17***	1.10	1.19***	1.14**	(1.04–1.25)
7,001–10,000	1.10*	1.04	1.11*	1.07	(0.97–1.18)
> 10,000	1	1	1	1	
Residence age 12 and at present					
Rural residents	1	1	1	1	
Rural to urban areas	1.24***	1.15***	1.23***	1.12*	(1.04–1.21)
Urban residents	1.32***	1.26***	1.30***	1.24***	(1.13–1.35)
***Social capital measures***					
Social trust	1	1		1	
Be cautious with others	2.12***	1.95***		1.88***	(1.76–2.01)
Social interaction	1	1		1	
Very few or never	1.34***	1.17***		1.18***	(1.08–1.28)
Social support	1	1		1	
Little or very little support	2.11***	1.83***		1.79***	(1.63–1.95)
***Other health covariate***					
Chronic illness^a^	1		1	1	
Yes	2.25***		2.26***	2.17***	(2.03–2.32)
Health-risk behaviours^b^					
Not a regular smoker	1		1	1	
Regular smoker	1.23**		1.12*	1.11	(0.98–1.26)
Not a regular alcohol drinker	1		1	1	
Regular alcohol drinker	1.70***		1.59***	1.58***	(1.36–1.85)

*Notes*: ^a^ Doctor diagnosed conditions including diabetes, high cholesterol, hypertension, cancers, goitre, epilepsy, arthritis, asthma or other chronic infections; ^b^ restricted to males due to very low rates among females.

Statistical significance *p < 0.05; **p < 0.01; *** *p* < 0.001.

**Table 4.** Odds Ratios relating to poor psychological health and social capital adjusting for individual characteristics, and health covariates

	Model 1 bivariate	Model 2 + social capital	Model 3 + health covariates	Model 4 adjusting for all covariates	95% Confidence Interval for Model 4
**Individual characteristics**					
Sex					
Males	1	1	1	1	
Females	1.16***	1.07***	1.15***	1.16***	(1.10–1.22)
Age in years					
20–29	1	1	1	1	
30–39	1.30***	1.26***	1.40***	1.35***	(1.23–1.47)
40–49	1.85***	1.73***	2.01***	1.92***	(1.75–2.10)
Marital status					
Married	1	1	1	1	
Not married	1.33***	1.11***	1.10***	1.13***	(1.07–1.18)
Separated, divorced, widowed	1.47***	1.38***	1.50***	1.36***	(1.22–1.52)
Income/month in Baht					
< 7,000	1.17***	1.08*	1.15***	1.11**	(1.03–1.17)
7,001–10,000	1.14***	0.96	0.99	0.97	(0.90–1.03)
> 10,000	1	1	1	1	
Residence					
Rural residents	1	1	1	1	
Rural to urban areas	1.13***	1.07*	1.18***	1.05*	(1.00–1.11)
Urban residents	1.20***	1.20***	1.25***	1.17***	(1.10–1.24)
**Social capital measures**					
Social trust	1	1		1	
Be cautious with others	2.77***	2.57***		2.52***	(2.41–2.64)
Social interaction	1	1		1	
Very few or never	1.38***	1.19***		1.21***	(1.14–1.28)
Social support	1	1		1	
Little or very little support	2.16***	1.83***		1.80***	(1.69–1.92)
**Other health covariates**					
Chronic illness^a^	1		1	1	
Yes	1.63***		1.63***	1.54***	(1.47–1.62)
Health-risk behaviours^b^					
Not a regular smoker	1		1	1	
Regular smoker	1.24***		1.16***	1.16***	(1.07–1.26)
Not a regular alcohol drinker	1		1	1	
Regular alcohol drinker	1.45***		1.37***	1.40***	(1.38–1.53)

*Notes*: ^a^ Doctor diagnosed conditions including diabetes, high cholesterol, hypertension, cancers, goitre, epilepsy, arthritis, asthma or other chronic infections; ^b^ restricted to males due to very low rates among females.

Statistical significance *p < 0.05; **p < 0.01; *** *p* < 0.001.

The fully adjusted Model 4 is the most informative and shows a strong association between low social trust and poor self-assessed overall health (OR = 1.88, 95% CI 1.76–2.01) and an even stronger association with poor psychological health (OR = 2.52, 95% CI 2.41–2.64). Perceived low social support was associated with poor self-assessed overall and poor psychological health (OR approximately 1.80 for both health outcomes); as well, chronic illness, regular smoking and regular alcohol drinking were all associated with both poor health outcomes.

## Discussion

In this study, we examine associations between social capital and self-assessed overall health and psychological health. After adjusting for potentially confounding variables, poor self-assessed health was significantly associated with having: chronic illness, low social trust, and low social support. Poor psychological health was also associated with having low social trust and having low social support. Female sex, older age, unpartnered, low income, urban residence, regular smoking, and regular alcohol consumption were also associated with poor self-assessed health and poor psychological health.

In our study, social capital was measured with social trust, participation, and support. However, in interpreting the results concerning social capital, one should keep in mind that the measures of social capital vary from one study to another (Petrou and Kupek, 2008). Despite the difficulty in comparing results of studies of social capital in different settings, countries with low levels of social capital generally have a high percentage of poor health reported as shown in a cross sectional study of 21 European countries among over 40,000 adults, where perceptions of social trust, membership, participation and voluntary work in civic organizations were used as social capital indicators (von dem Knesebeck et al., 2005).

Studies in East Asia have found strong evidence of social support and networks in more than 20 villages in rural China, where mistrust is more powerfully associated with worse mental health (Wang et al., 2009). Another study, also in rural China, has found a positive association between social capital, especially social trust, and subjective well-being (Yip et al., 2007) – the importance of intergenerational social capital in rural areas noting positive effects on both adolescents and elderly people (De Souza and Grundy, 2007).

Other related analyses from this Thai Health-Risk Transition project have confirmed that rural residents were more likely to interact with family and friends; in contrast, among urban residents social interaction and support was more likely to come from work colleagues (Yiengprugsawan et al., 2009). These work related contacts could be important channels for strengthening social capital in urban populations. Indeed, two Finnish studies of 15,000 public sector and local government employees found exposure to low social capital at work could be detrimental to the health of employees (Liukkonen et al., 2004; Oksanen et al., 2008).

Another type of social trust which was not explored in our study and which is worth future investigation in middle-income settings was political or vertical trust. Some studies based on populations in Europe have found low political trust to be significantly and positively associated with poor self-rated heath (OR = 2.1 to 2.4 for males and OR = 1.6 to 1.9 for females) (Mohseni and Lindstrom, 2008) and poor psychological health (OR = 1.4 to 1.6 for males and OR = 1.4 to1.7 for females) after adjusting for socio-demographic characteristics and horizontal trust (Lindstrom and Mohseni, 2009).

We note a possible limitation of our study relates to the temporal relationship between social capital and health. As this and most of the earlier studies on social capital and health have been based on cross-sectional data, the direction of the relationship between these factors is uncertain with a possibility of reverse causation between social capital and health. Longitudinal study could provide more insight into the possible pathway and this has now been done in at least one setting in Britain, showing that low social capital led to poor health outcomes over time (Giordano and Lindstrom, 2010). The cohort we studied here is under longitudinal observation to enable such assessment in the future. Based on our findings shown here, we concluded that social capital in Thailand by standard measures was positively associated with both self-assessed overall health and psychological health. Our findings may assist in the development of policy and programmes which are effective in promoting social trust and social networking which could lead to positive health and well-being in the Thai context.
